# Endocrine effect of phthalate metabolites and a butterfly effect of prenatal exposure to androgens on qualitative aspects of female sexual response- an initial survey

**DOI:** 10.1038/s41443-024-00919-1

**Published:** 2024-05-28

**Authors:** Branislav Kolena, Henrieta Hlisníková, Miroslava Nagyová, Katarína Orendáčová, Mária Vondráková, Ida Petrovičová, Miloš Mlynček, Petr Weiss, James G. Pfaus

**Affiliations:** 1 Department of Zoology and Anthropology, Constantine the Philosopher University, Nitra, Slovakia; 2 Department of Nursing, Constantine the Philosopher University, Nitra, Slovakia; 3 Department of Psychology, Charles University, Prague, Czech Republic; 4 Deaprtment of Psychology, Comenius University, Bratislava, Slovakia; 5 Department of Psychology and Life Sciences, Faculty of Humanities, Charles University, Prague, Czech Republic; 6 Center for Sexual Health and Intervention, Czech National Institute of Mental Health, Klecany, Czech Republic

**Keywords:** Risk factors, Human behaviour

## Abstract

There is growing evidence that endocrine disruptive chemicals have deleterious effects on sexual and reproductive function. To examine subjective sexual functions in human females and their relationship to postnatal phthalate exposure and perinatal androgenization, a Sexuality Score (SS) was established from a first-stage survey questionnaire of subjective sexual function filled out by female university students (*n* = 68; average age 25.23 ± 5.17 years; rural 25.51 ± 6.74 vs. urban 25.85 ± 1.43 years). Seventeen phthalate metabolites in urine samples were analyzed by high‐performance liquid chromatography (HPLC) and tandem mass spectrometry (MS/MS). Females were also assessed for the 2D:4D digit ratio as an index of perinatal androgenization. The mean age of menarche was 12.82 ± 1.35 years (rural 12.59 ± 1.39 vs. urban 13.18 ± 1.27; *p* = 0.01). The mean age at first sexual intercourse was 14.88 ± 6.89 years (rural 14.62 ± 7.20 vs. urban 15.24 ± 6.55), and as the age of first sexual intercourse increases, the SS score tends to increase as well, albeit moderately (r = 0.25, *p* = 0.037). Mono‐iso‐butyl phthalate, mono(2‐ethyl‐5‐carboxypentyl) phthalate, mono(hydroxy‐n‐butyl) phthalate, mono(2‐ethyl‐5‐oxohexyl) phthalate (p ≤ 0.05) and mono(2-carboxymethylhexyl) phthalate (p ≤ 0.01) were negatively associated with SS. A compounding butterfly effect of prenatal exposure to androgens was observed with disruptive effects of mono(2‐ethyl‐5‐oxohexyl) phthalate and mono(2‐ethyl‐5‐carboxypentyl) phthalate on sexual function. Exposure to phthalates in adult females may lead to disruption of subjective sexual function, especially concerning sexual desire and sexual satisfaction, and perinatal androgenization could augment these effects.

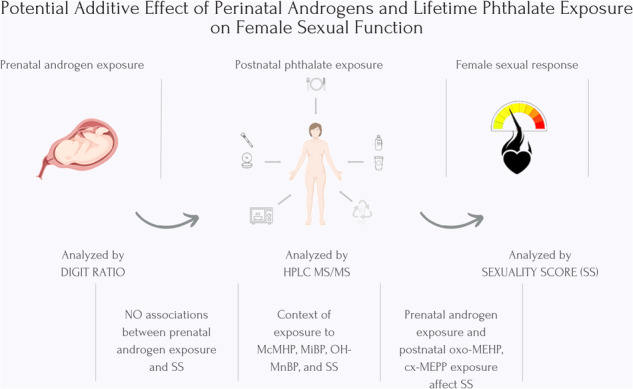

## Introduction

Our understanding of human sexual development remains incomplete regarding the molecular events involved. Genetic control, primarily influenced by sex chromosomal differences, plays a pivotal role in initiating sexual development. This process triggers gonadal development, followed by the differentiation of observable traits, regulated by hormonal activity. Sex steroids, which exert both lasting and temporary effects, are also of paramount importance. Permanent changes in phenotype are induced through the genetic control of downstream genes, with androgens, acting through the androgen receptor, being key elements in this differentiation process. The specificity of androgen action appears to be a time-sensitive process, where the androgen receptor collaborates with various metabolites and cofactors to modulate cellular responses, thereby permanently shaping an individual’s phenotype [1].

Endocrine disrupting chemicals (EDCs) found in the environment (including the food chain) mimic, block, or otherwise interfere with steroid hormone actions in a variety of species [2–4]. Women’s reproductive health relies on the synchronized functioning of a network of endocrine signals which play a crucial role not only in ensuring successful procreation but also exerting diverse effects on the body. These effects extend beyond secondary reproductive tissues to include bone, brain, and the cardiovascular system [5]. Disruption can occur both during the critical perinatal period for sexual differentiation and brain/body organization, and later in life during adolescence and across the lifespan to affect the activation of sexual behavior and reproductive functions. Animal studies show that exposure to EDCs (such as polychlorinated biphenyls, bisphenol A (BPA), plasticizers- including phthalates, etc) have demasculinizing effects on male reproductive organs, diminish semen quality, and impair sexual behavior due to either estrogenic or antiandrogenic actions exerted in utero and/or in adulthood [3, 6–11]. Studies in female rodents have shown that perinatal exposure to BPA or phthalate/adipate esters (such as di-n-butyl phthalate (DBP), diisononyl phthalate (DINP), and Di(2-ethylhexyl) adipate (DEHA)), reduce measures of proceptive and receptive sexual behaviors, such as solicitations and lordosis, along with reductions in estrogen receptor levels in the hypothalamus [12, 13].

Phthalates are used in the production of plastics and are found extensively as waste byproducts. Although they are metabolized in the human body and excreted, in urine within 24 to 48 hours of exposure [14], chronic exposure has the potential to reach a point where the rate of intake and elimination balances out. Some EDCs have been classified specifically as reproductive toxins based on their ability to interfere with normal reproductive function and hormone signaling [15]. For example, phthalates induce negative feedback on ovarian function by activating estrogen receptors (ERα and ERβ) and by inhibiting the androgen receptor (AR) as initial molecular events. Additionally, it is recognized that primary and secondary metabolites of phthalates elicit distinct effects at the molecular level in comparison to the parental compounds [16]. Exposure is thus a risk to the survival of both terrestrial and aquatic species, and given the widespread use of plastics and other phthalate-containing substances, this problem is likely to be much more pronounced in the future. Due to the non-monotonic effect on biological systems, instead of a consistent increase or decrease in response as the dose rises, the effects may fluctuate, plateau, or even reverse at higher concentrations.

Disruptions in sexual and reproductive functions by EDCs have been reported in studies involving humans. For example, exposure to BPA and its metabolites impair spermatogenesis and reduce male sexual desire and ejaculatory strength [17, 18]. In animal studies, exposure of female rats to bisphenols disrupts the normal function of the hypothalamic-pituitary-gonadal (HPG) axis and reduces ovarian output of 17-β-estradiol (E2), progesterone (P4), and testosterone (T), which can lead to disruptions of ovulation, including infertility and polycystic ovary syndrome, and potentially decrease sexual arousal and desire [19]. Phthalate exposure also disrupts the HPG axis and ovarian function [20] and is associated with earlier thelarche (Tanner stage growth changes) but delayed pubarche [21], suggesting a disruption in pubertal transition [22]. To date, one study has reported that exposure to specific metabolites of the phthalate DEHP, such as mono-2-ethyl-5-hydroxyhexyl phthalate (OH-MEHP) and oxo-MEHP, is associated with decreased sexual desire in premenopausal women [23].

The effects of perinatal androgenization on specific parts of the human body are not completely understood. This delicate equilibrium can be disrupted by external factors, and one such disruptor is exposure to phthalates. Understanding androgenization during female fetal development is crucial not only for comprehending the natural processes that lead to sexual differentiation but also for addressing clinical conditions that may arise when this balance is disrupted [21, 24]. The digit ratio, specifically the lengths of the second (index) and fourth (ring) fingers, has garnered attention as marker of prenatal androgenization. A lower digit ratio (a longer fourth finger relative to the second) indicate higher prenatal androgen exposure, while a higher digit ratio (a shorter fourth finger relative to the second) suggests lower androgen exposure [25, 26]. The digit ratio remains fairly constant from birth or later infancy onward and there is no observable correlation between digit ratio and levels of adult sex hormones. Instead, it seems to be linked to androgen sensitivity rather than the actual concentration of androgens. Studies have linked variations in digit ratio to a range of behavioral (e.g. assertiveness, aggression, anxiety), physiological (obesity, handed preference, sperm account), and health outcomes [reviewed in 26].

The present study examined the degree of phthalate exposure on the global sexual function of a sample of reproductive-age females in Slovakia, with special emphasis on demographic and anthropometric measures, and level of perinatal androgenization estimated from 2D:4D digit ratios, as potential confounding factors.

## Method

### Participants

Participants were 68 adult female university students from Slovakia. The recruitment period for this study started on February 14, 2021, and ended on December 28, 2021.The eligibility criteria were age ≥18 years old, regular menstrual cycles, not using hormonal contraception and not being pregnant, no endocrine disorders, being sexually active and without pain during sexual intercourse. Participants were asked to estimate their ovulation day from the first day of their menstruation, and to come in during their ovulatory phase to complete questionnaires and provide first morning urine samples and anthropomorphic measures. The study was approved by the Ethics Committee of the Faculty Hospital in Nitra, Slovakia, and complied with the principles of the Helsinki Declaration regarding human experimentation. Participants signed informed consent before the study, and it was possible to withdraw participation at any time during the study.

### Assessment of subjective female sexual response

A non-validated “Slovak-language general questionnaire of subjective sexual function” was developed to assess global sexual function (the Supplementary material, Supplement 1). The questionnaire contained 8 questions with multiple-choice responses that assess sexual function associated with desire, arousal, orgasm, sexual satisfaction during or after solo or partnered sexual activities. Participants chose the option that best described their situation in the following questions:

*A. Please indicate the frequency of your sexual intercourse in the last month*.

*B. How often have you masturbated in the last month?*

*C. “My current sexual desire (excitement) is strong.”*

*D. How often do you have sexual fantasies?*

*E. Please express your satisfaction/dissatisfaction with your sexual arousal*.

*F. How often do you have a desire for sexual satisfaction?*

*G. Are you interested in books, movies, websites, music, or drawings with a sexual theme?*

*H. If you were to compare yourself to your peers, how intense is your sexual desire?*

Each item was associated with a percentage value corresponding to the degree of gratification of the participant and had a total score of 1. According to the formula, answers were scored on an Likert scale, where we divided the answer option by the number of answer options for a particular question. For questions 1 and 2, there were eight options. For questions 3–8, there were five options. A total Sexuality Score (SS) was subsequently calculated as the sum of the obtained values from questions 1–8.

### Anthropometric measurements

All anthropometric measures were performed by a trained researcher using a standardized protocol. Height, weight, waist and hip circumference were measured to the nearest 0.5 cm, 0.1 kg, and 0.5 cm, respectively. BMI was calculated as (weight (kg)/height (m)^2^). Body composition (weight, body fat percentage, muscle mass percentage, and visceral fat level) was estimated by The Omron BF510 (Kyoto, Japan). Waist-to-height ratio (WHtR), waist-to-hip ratio (WHR), fat mass index (FMI), and fat-free mass index (FFMI) were calculated. The length of the right index (2D) and ring (4D) fingers on the dominant hand was taken using a digital calliper (POWER-FIX PROFI, model Z22855) with an accuracy of 0.05 mm [26]. In order to minimize the measurement error, all measurements were performed three times and the results presented as the average of these values.

### Urinary analyses of phthalates

Females provided first-morning urine samples (2 × 2 mL). The description of qualitative and quantitative analysis of phthalate metabolites is described elsewhere [27]. We used high‐performance liquid chromatography (HPLC) and tandem mass spectrometry (MS/MS) (Infinity 1260 and 6410 triplequad, Agilent, Santa Clara, CA, USA) to analyze the urinary concentration of phthalate metanbolites by previously published on-line methods by HPLC‐MS/MS [28, 29]. Phthalates and their metabolites are shown in Table 1.

**Table 1:** Primary and secondary metabolites of analyzed monoester of phthalates.

Phthalate diester	Primary metabolite	Secondary metabolite
DMP	MMP	–
DEP	MEP	–
DiBP	MiBP	–
DnBP	MnBP	OH-MnBP
BBzP	MBzP	–
DPeP	MnPeP	–
DCHP	MCHP	–
DEHP	MEHP	OH-MEHP
		oxo-MEHP
		cx-MEPP
		McMHP
DiNP	–	OH-MiNP
		oxo-MiNP
		cx-MiNP

*BBzP* benzylbutyl phthalate, *cx‐MEPP* mono(2‐ethyl‐5‐carboxypentyl) phthalate, *cx‐MiNP* mono-carboxy-isononyl phthalate, *DEHP* di(2-ethylhexyl) phthalate, *DEP* diethyl phthalate, *DCHP* dicyclohexyl phthalate, *DiBP* di-iso-butyl phthalate, *DiNP* di-iso-nonyl phthalate, *DMP* dimethyl phthalate, *DnBP* di-iso-butyl phthalate, *DPeP* dipentyl phthalate, *MBzP* monobenzyl phthalate, *MEP* monoethyl phthalate, *MCHP* monocyclohexyl phthalate, *MiBP* mono‐iso‐butyl phthalate, *MiNP* mono‐iso‐nonyl phthalate, *MMP* monomethyl phthalate, *MnBP* mono‐n‐butyl phthalate, *MnOP* mono‐n‐octyl phthalate, *MnPeP* mono‐n‐pentyl phthalate, *OH‐MEHP* mono(2‐ethyl‐5‐hydroxyhexyl) phthalate, *OH‐MiBP* mono(hydroxy‐iso‐ butyl) phthalate, *OH‐MiNP* mono-hydroxy-isononyl phthalate, *OH‐MnBP* mono(hydroxy‐n‐butyl) phthalate, *oxo‐MEHP* mono(2‐ethyl‐5‐oxohexyl) phthalate, *oxo‐MiNP* mono-oxo-isononyl phthalate, *McMHP* Mono(2-carboxymethylhexyl) phthalate.

The analysis was performed in the Physiological Analytical Laboratory, which participated in the HBM4EU QA/QC programme. Its successful performance resulted in its qualification as an HBM4EU laboratory for the analysis of phthalate metabolites in human urine. While testing, satisfactory Z-scores were obtained for these compounds ranging from −1.1 to 0.7. The interlaboratory test conditions for a successful passing were the Z scores ≤ |2 | [30]. Internal quality control was performed by analyses of 2 control materials (a mixture of urine samples) with known lower and higher concentrations. The limits of quantification (LOQ) were estimated between 1 and 2.5 ng/mL.

### Statistical analyses

Mean urinary phthalate metabolite concentrations, anthropometry, and SS data were calculated from each participant. The Shapiro–Wilk test was used as a test of normality. Correlations between the values obtained in the questions of the sexual questionnaire and the concentrations of metabolites were examined by Spearman’s correlation. Correlations between phthalate metabolite concentrations and total SS were examined by Pearson correlation analysis, in which urinary phthalate metabolite concentrations were log-transformed to base 10. The nonparametric Mann–Whitney U (Wilcoxon rank‐sum) test and Kruskal–Wallis one‐way analysis of variance were used to analyze differences between two or more groups in the baseline characteristics of females associated with phthalate metabolites. A one-way analysis of variance (ANOVA) was used to determine whether there were any statistically significant differences between the interquartile distribution of phthalate metabolites and SS. Pearson correlation analysis, unpaired t-test, and ANOVA were used to determine confounding variables from individuals’ baseline characteristics. We analyzed the following continuous variables: age, BMI, WHR, WHtR, % body fat and muscle, age of first menstrual period, age of first sexual intercourse; and nominal variables: place of residence, current relationship, and boy-toy preferences during the childhood. Hierarchical multiple regression modeling was used to examine the relationship between phthalates metabolites and the length of the second digit (2D), the fourth digit (4D) and digit ratio (2D:4D) (independent variables) and SS (dependent variable). All statistical analyses were performed using Statistica 7 (StatSoft), and IBM SPSS Statistics (version 21.0; SPSS Inc., Chicago, IL, USA). Differences were statistically significant when p ≤ 0.05. Effect size estimates in correlation analyses are represented by *r* (correlation), *β* (regression) and *ε²* (Kruskal-Wallis test). The sample size calculation was performed using G*Power (version 3.1) [31]. We made the correlation matrices in R using the R package “metan”.

## Results

### Demographic and anthropometric characteristics

Table 2 shows the baseline characteristics of the cohort (*n* = 68).

**Table 2:** Baseline characteristics of the participants (means ± SD).

	Participants (n = 68)	Rural (n = 39)	Urban (n = 29)	p
Age (years)	25.23 ± 5.17	25.51 ± 6.74	25.85 ± 1.43	0.487
Age of menarche (years)	12.82 ± 1.35	12.59 ± 1.39	13.18 ± 1.27	**0.001**
Age of first sexual intercourse	14.88 ± 6.89	14.62 ± 7.20	15.24 ± 6.55	0.710
Body height (cm)	167.34 ± 5.46	167.32 ± 5.44	167.36 ± 5.59	0.891
Body weight (kg)	65.32 ± 14.41	65.62 ± 14.42	64.91 ± 14.64	0.809
Waist circumference (cm)	83.36 ± 13.84	83.08 ± 13.54	83.74 ± 14.47	0.877
Hip circumference (cm)	100.30 ± 10.23	100.57 ± 9.64	99.94 ± 11.14	0.352
BMI (kg/m^2^)	23.26 ± 4.77	23.29 ± 4.15	23.22 ± 5.58	0.539
WHR	0.83 ± 0.10	0.83 ± 0.11	0.84 ± 0.08	0.857
WHtR	0.50 ± 0.08	0.50 ± 0.07	0.50 ± 0.09	0.936
Fat (%)	33.29 ± 8.20	33.39 ± 8.13	33.16 ± 8.44	0.805
Muscle (%)	27.11 ± 4.02	26.44 ± 4.45	27.98 ± 3.24	0.330
2D dx. (cm)	6.78 ± 0.83	6.68 ± 0.80	6.91 ± 0.87	0.260
4D dx. (cm)	6.75 ± 0.84	6.67 ± 0.92	6.85 ± 0.72	0.820
Digit Ratio (2D:4D dx.)	1.01 ± 0.10	1.01 ± 0.12	1.01 ± 0.08	0.199
Score of sexuality	12.93 ± 5.02	13.00 ± 4.79	12.83 ± 5.40	0.749

2D The length of the index finger on the right hand, 4D The length of the ring finger on the right hand; nonparametric Mann–Whitney U (Wilcoxon rank‐sum) test was used to analyze differences between two groups

Statistically significant *p*-values are in bold.

*BMI* Body Mass Index, *WHR* Waist to Hip Ratio, *WHtR* Waist to Height ratio.

The mean age of the sample was 25.23 ± 5.17 years (rural 25.51 ± 6.74 vs. urban 25.85 ± 1.43; *p* = 0.487). We did not observe statistically significant differences between the percentage of participants who came from urban or rural areas (urban 42.65% vs. rural 57.35%). However, the mean age of menarche was 12.82 ± 1.35 years (rural 12.59 ± 1.39 vs. urban 13.18 ± 1.27), and this difference by place of residence was statistically significant (*p* = 0.01). The onset of menarche in 33.82% of females (*n* = 23) occurred at the age of 12 years, then at the age of 13 years (*n* = 18; 26.47%), and 14 years (*n* = 10; 14.71%). In six individuals, the onset of menarche was observed at both 11 years and 15 years (7.35%). In the other cases, the age of first menstruation was 15 years or older (*n* = 4; 5.88%) and 10 years (*n* = 3; 4.41%). The mean age at first sexual intercourse was 14.88 ± 6.89 years (rural 14.62 ± 7.20 vs. urban 15.24 ± 6.55; *p* = 0.710), and it correlated positively with SS (r = 0.24; *p* = 0.05). Body weight also correlated positively with SS (r = 0.25, *p* = 0.037).

### Urinary phthalate metabolite concentrations

Descriptive statistics of concentration of phthalate metabolites (ng/mL) in our participants are shown in Table 3.

**Table 3:** Descriptive statistics of phthalate metabolite concentrations (ng/mL).

Variable	Mean	Median	Min	Max	percentile		SD	CI	
					25	95		–95	+95
MMP	1.59	1.25	1.25	5.67	1.25	1.25	0.92	0.79	1.11
MEP	176.75	43.42	4.20	6799.42	19.13	85.36	820.98	702.44	988.01
MiBP	41.08	30.32	1.77	313.56	16.25	47.19	46.86	40.09	56.39
OH-MiBP	6.76	5.47	0.70	39.44	3.46	8.17	5.63	4.82	6.77
MnBP	71.03	57.28	1.77	530.27	35.87	96.80	70.41	60.24	84.74
OH-MnBP	15.43	11.70	0.70	88.02	5.99	18.97	14.81	12.67	17.82
MBzP	2.05	1.21	0.50	16.97	0.50	2.38	2.97	2.54	3.58
MnPeP	1.25	1.25	1.25	1.25	1.25	1.25	0.00	0.00	0.00
MCHP	1.38	1.38	0.50	7.92	0.50	1.92	1.17	1.01	1.41
MEHP	4.64	3.23	1.00	50.30	1.41	5.04	6.59	5.64	7.93
OH-MEHP	15.76	9.78	1.41	210.32	6.91	14.98	27.31	23.37	32.87
oxo-MEHP	8.88	6.33	0.70	90.73	4.10	10.06	11.27	9.64	13.56
cx-MEPP	15.12	10.95	0.70	154.48	7.13	16.77	19.78	16.92	23.80
McMHP	5.72	4.57	1.25	26.13	3.55	6.26	4.24	3.63	5.10
OH-MiNP	12.32	10.97	1.06	59.42	4.50	15.31	9.66	8.27	11.63
oxo-MiNP	3.10	2.56	0.75	14.52	1.29	4.44	2.48	2.13	2.99
cx-MiNP	6.09	4.64	0.70	56.48	3.08	6.96	7.09	6.07	8.54

*cx‐MEPP* mono(2‐ethyl‐5‐carboxypentyl) phthalate, *cx‐MiNP* mono-carboxy-isononyl phthalate, *MBzP* monobenzyl phthalate, *MCHP* monocyclohexyl phthalate, *MEP* monoethyl phthalate, *MiBP* mono‐iso‐butyl phthalate, *MiNP* mono‐iso‐nonyl phthalate, *MMP* monomethyl phthalate, *MnBP* mono‐n‐butyl phthalate, *MnOP* mono‐n‐octyl phthalate, *MnPeP* mono‐n‐pentyl phthalate, *OH‐MEHP* mono(2‐ethyl‐5‐hydroxyhexyl) phthalate, *OH‐MiBP* mono(hydroxy‐iso‐ butyl) phthalate, *OH‐MiNP* mono-hydroxy-isononyl phthalate, *OH‐MnBP* mono(hydroxy‐n‐butyl) phthalate, *oxo‐MEHP* mono(2‐ethyl‐5‐oxohexyl) phthalate, *oxo‐MiNP* mono-oxo-isononyl phthalate, *McMHP* Mono(2-carboxymethylhexyl) phthalate.

**Fig. 1:**
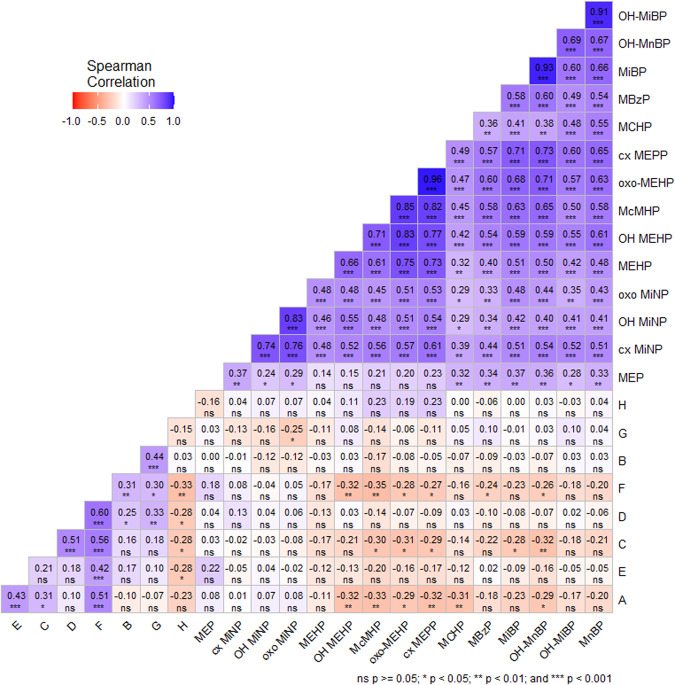
Intercorrelations among the different phthalate concentrations and specific questions of general questionnaire of subjective sexual function. **A** Please indicate the frequency of your sexual intercourse in the last month; (**B**) how often have you masturbated in the last month?; (**C**) “My current sexual desire (excitement) is strong.”; (**D**) how often do you have sexual fantasies?; (**E**) please express your satisfaction/dissatisfaction with your sexual arousal.; (**F**) how often do you have a desire for sexual satisfaction?; (**G**) are you interested in books, movies, websites, music, or drawings with a sexual theme?; (**H**) If you were to compare yourself to your peers, how intense is your sexual desire?; cx‐MEPP mono(2‐ethyl‐5‐carboxypentyl) phthalate, cx‐MiNP mono-carboxy-isononyl phthalate, MBzP monobenzyl phthalate, MCHP monocyclohexyl phthalate, MEP monoethyl phthalate, MiBP mono‐iso‐butyl phthalate, MiNP mono‐iso‐nonyl phthalate, MMP monomethyl phthalate, MnBP mono‐n‐butyl phthalate, MnOP mono‐n‐octyl phthalate, MnPeP mono‐n‐pentyl phthalate, OH‐MEHP mono(2‐ethyl‐5‐hydroxyhexyl) phthalate, OH‐MiBP mono(hydroxy‐iso‐ butyl) phthalate, OH‐MiNP mono-hydroxy-isononyl phthalate, OH‐MnBP mono(hydroxy‐n‐butyl) phthalate, oxo‐MEHP mono(2‐ethyl‐5‐oxohexyl) phthalate, oxo‐MiNP mono-oxo-isononyl phthalate, McMHP Mono(2-carboxymethylhexyl) phthalate; * p < 0.05, ** p < 0.01, *** p < 0.001.

The urinary concentrations of detected phthalate metabolites were above the LOQ in 100% of samples for MEP and OH MEHP, 98.5% for MiBP, MnBP, OH-MnBP, oxo-MEHP, cx-MEPP, oxo-MiNP, cx-MiNP, 97.1% for OH-MiBP, 91.2% for McMHP, 75% for oxo-MiNP, 72.1% for MEHP, 55.9% for MBzP and MCHP and 13.2% for MMP. It is important to note that many phthalate metabolites were found in each urine sample. The intercorrelations among the different phthalate metabolite concentrations are shown in Fig. 1.

### Prenatal markers of androgenization, phthalate metabolites concentration and general questionnaire of subjective sexual function

**Fig. 2:**
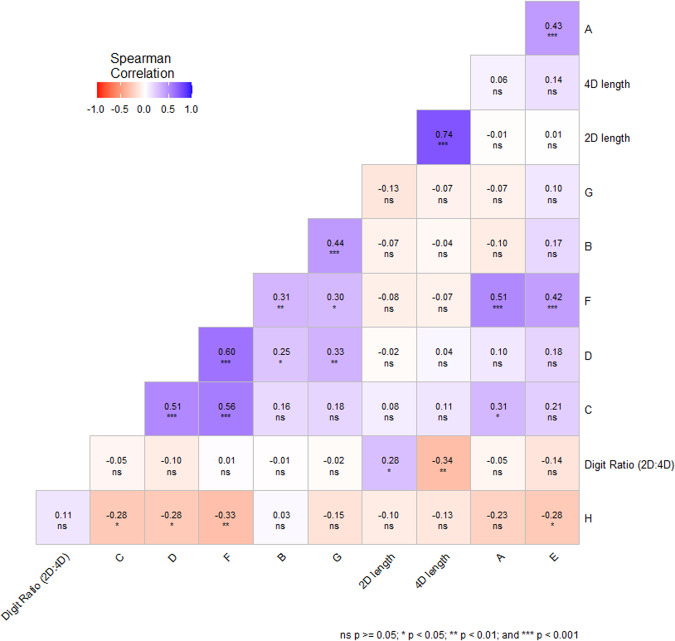
Prenatal markers of androgenization and their correlation with specific questions of general questionnaire of subjective sexual function. **A** Please indicate the frequency of your sexual intercourse in the last month; (**B**) how often have you masturbated in the last month?; (**C**) “My current sexual desire (excitement) is strong.”; (**D**) how often do you have sexual fantasies?; (**E**) please express your satisfaction/dissatisfaction with your sexual arousal.; (**F**) How often do you have a desire for sexual satisfaction?; (**G**) are you interested in books, movies, websites, music, or drawings with a sexual theme?; (**H**) if you were to compare yourself to your peers, how intense is your sexual desire?; 2D length—The length of the index finger on the right hand, 4D length—The length of the ring finger on the right hand; Digit Ratio (2D:4D)—The ratio of the lengths of the index and ring finger on the right hand.

Figure 2 shows a “heat map” breakdown of prenatal markers of androgenization and their correlation with specific questions of general questionnaire of subjective sexual function. We did not observe any associations between these variables. However, correlation analysis revealed positive and negative associations among the questionnaire items A-H, which suggest the utility of these items for assessing the sexual score in the next steps. In addition, (based on the results of Fig. 1, which shows a “heat map” breakdown of phthalate concentrations and their correlation with specific questions of the general questionnaire of subjective sexual function) we proceeded to evaluate the impact of phthalates on sexual scores.

Questions A (frequency of sexual intercourse), C (current level of sexual desire and excitement), F (desire for sexual satisfaction), and G (interest in visual sexual stimuli) showed significant negative correlations with McMHP, oxo-MEHP, and cx MEPP in 3 of the 4 questions. Other individual phthalates also correlated negatively with these questions.

### Phthalate metabolites concentration and Sexuality Score

In the next step, with the exception of metabolites MEP a cx-MiNP, which correlated positively, we observed a negative correlation in the case of the other metabolites with the SS, but only in the case of metabolites McMHP (r = –0.39, p ≤ 0.01) and MiBP (r = –0.31, p ≤ 0.05), OH-MnBP (r = –0.31, p ≤ 0.05), oxo MEHP (r = –0.27, p ≤ 0.05), cx MEPP (r = –0.29, p ≤ 0.05) this negative correlation was found to be statistically significant.

**Fig. 3:**
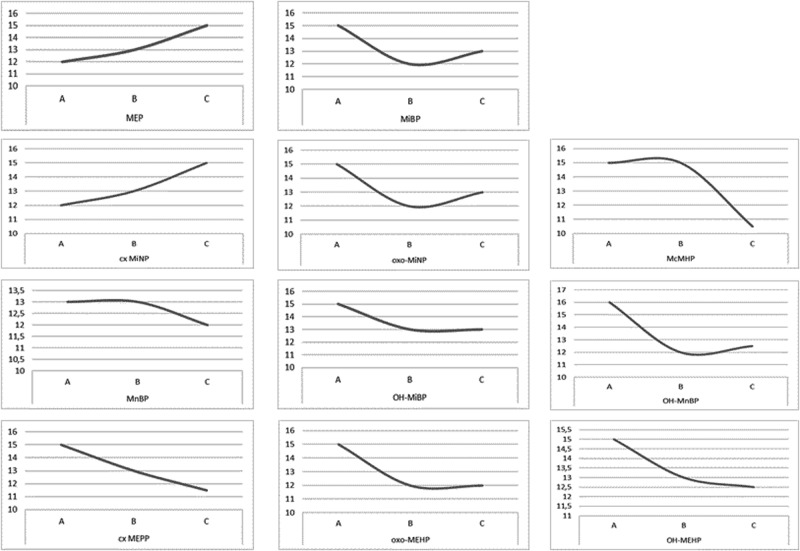
Trend of phthalate metabolites distribution and potential effect on the sexuality score. x-axis: **A** First tercile of phthalate metabolite concentration (ng/mL); (**B**) second tercile of phthalate metabolite concentration (ng/mL); (**C**) third tercile of phthalate metabolite concentration (ng/mL); MEP monoethyl phthalate, MiBP mono‐iso‐butyl phthalate, cx‐MiNP mono-carboxy-isononyl phthalate, oxo‐MiNP mono-oxo-isononyl phthalate, McMHP Mono(2-carboxymethylhexyl) phthalate, MnBP mono‐n‐butyl phthalate, OH‐MiBP mono(hydroxy‐iso‐ butyl) phthalate, OH‐MnBP mono(hydroxy‐n‐butyl) phthalate, cx‐MEPP mono(2‐ethyl‐5‐carboxypentyl) phthalate, oxo‐MEHP mono(2‐ethyl‐5‐oxohexyl) phthalate, OH‐MEHP mono(2‐ethyl‐5‐hydroxyhexyl) phthalate, y-axis: sexuality score.

To observe the trend of phthalate metabolites distribution and their potential effect on SS, we divided the group across the interquartile range only in phthalate metabolites whose concentrations were determined above the detection limit in more than 70% of the analyzed samples. In the case of MiBP, the highest SS was achieved by individuals with the lowest concentration in the first quartile (SS = 15; MiBP mean 12.124 ± 0.961 ng/mL, median 12.368 ng/mL), compared to the second (SS = 12; MiBP mean 30.282 ± 1.828 ng/mL, median 30.558 ng/mL; *p* = 0.052) and third quartiles (SS = 13; MiBP mean 82.653 ± 13.562 ng/mL, medain 56.594 ng/mL; *p* = 0.036). The same pattern was observed with OH-MnBP in the first quartile (SS = 16; OH-MnBP mean 4.662 ± 0.389 ng/mL, median 5.061 ng/mL) compared to the second (SS = 12; OH-MnBP mean 12.118 ± 0.671 ng/mL, median 12.104 ng/mL; *p* = 0.017) and third quartiles (SS = 12.5; OH-MnBP mean 30.157 ± 3.807 ng/mL, median 22.244 ng/mL; *p* = 0.052). No statistical difference were observed among the other metabolites, although the nonlinear, linear and inverse relationships between metabolites and total SS are worth mentioning (Fig. 3).

### Association between prenatal androgenization (2D:4D ratios) and postnatal phthalate metabolites concentration

**Fig. 4:**
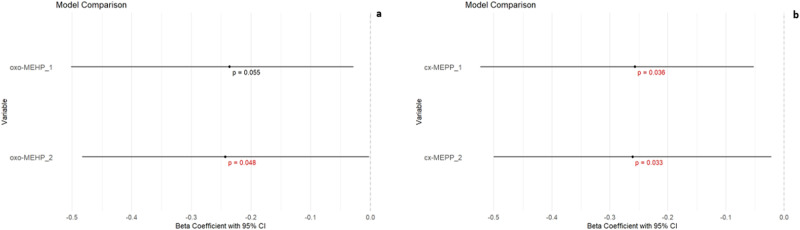
Relationship between phthalate metabolites and sexuality score: adjusted for 2D:4D digit ratio. **a** oxo-MEHP_1—model: mono(2‐ethyl‐5‐oxohexyl) phthalate;pxp-MEHP_2—model: mono(2‐ethyl‐5‐oxohexyl) phthalate adjusted for Digit Ratio; (**b**) cx-MEPP_1—model: mono(2‐ethyl‐5‐carboxypentyl) phthalate; cx-MEPP_2—model: mono(2‐ethyl‐5‐carboxypentyl) phthalate adjusted for Digit Ratio.

Figure 4 shows only the results of hierarchical linear regression of the statistical significant association between total SS and two phthalate metabolites, oxo-MEHP and cx-MEPP, adjusted for digit ratio (2D:4D). The statistical significance and β-value of these two metabolites increased after adjustment for the 2D:4D ratio in the regression model Complete results can be found in the Supplementary Material (Supplementary 2, Table S1–S3).

## Discussion

We live in the age of plastics. During the production of plastics, a large number of plasticizers, including phthalates, are used. This research indicates that Slovak women of reproductive age, residing in both rural and urban regions, encounter exposure to phthalates. This exposure appears to have adverse effects on subjective evaluations of sexual desire and satisfaction. The phthalate metabolites of DEHP and DiBP were associated with lower scores overall on the SS and in particular on questions relating to the frequency of sexual intercourse, current level of sexual desire and excitement, desire for sexual satisfaction, and interest in visual sexual stimuli. Phthalate metabolite concentration was not correlated with lower scores on questions regarding frequency of masturbation, sexual fantasies, or how their perceived desire compared to that of their peers. Finally, a small but potentially important increase in effect size was detected for the effect of DEHP metabolites on these measures when the 2D:4D ratio was factored in. This suggests that early perinatal androgenization may act like a butterfly effect (Fig. 4) as a potential confounding factor that makes women more susceptible to the effects of certain phthalate metabolites in adulthood.

The exact mechanisms by which phthalates disrupt sexual function is currently unclear. Phthalates are used as plasticizers in plastic products [32], which mean that people are exposed to those plasticizers daily. Phthalate diesters and their metabolites are found in soil, drinking water, air, dust, and foods; thus, people are exposed to phthalates by food intake, inhalation, intravenously, and through dermal contact [21], and exposure is ubiquitous. Low doses of phthalates have estrogenic and/or anti-androgenic effects during perinatal, peripubertal, and adult life, which have been studied more extensively in rodent models [21, 24]. As EDCs, phthalates and their metabolites induce gonadal dysregulation in both males and females, and were linked with infertility, testicular dysgenesis syndrome (known as “phthalate syndrome” in animal models), ovarian failure, gonadal cancers, acceleration or delay in puberty (depending on when and how phthalates disrupt the HPG axis), and dysfunctions of pregnancy [3, 21, 24]. Some of these effects occur directly in testicular or ovarian cells, or in neurons in the hypothalamus and gonadotrophic cells of the pituitary, and lead to decreases in E2, T, and P4. Phthalates can also interfere with pituitary thyroid stimulating hormone release, leading to decreased secretion of thyroid hormones, and alter the hypothalamic-pituitary-adrenal axis, leading to decreased adrenal output of glucocorticoids [24].

Prenatal phthalate exposure can induce the onset of neurodevelopmental disorders, such as ADHD, ASD, and cognitive and behavioral disorders [24]. Unfortunately, we were unable to assess phthalate exposure during the prenatal development of the individuals in our study. However, considering the ongoing elimination of phthalates, especially DEHP, from production due to recognized toxicity and regulatory measures (or their substitution with alternatives) [33], which varies uniquely in each country [34, 35], we can reasonably infer that the participants in our study experienced significantly higher concentrations of phthalates during their childhood compared to the present time.

The results of the present study suggest that exposure to phthalates is also involved in the subjective experience of decreased sexual desire and sexual satisfaction of women, an effect that could result from decreased ovarian output of androgens, or from antiandrogenic effects on nuclear or membrane bound androgen receptors. This could be studied explicitly in rodent models using sophisticated behavioral paradigms that have predictive validity for human sexual response [36, 37]. In addition, there is evidence indicating that phthalates might exhibit non-monotonic effects [38, 39], which was also noted in our study for metabolites MEP, MiBP, cx MiNP, oxo MiNP, McMHP, MnBP, OH-MiBP, OH-MnBP, cx-MEPP, oxo-MEHP and OH-MEHP (Fig. 3). The non-monotonic effect of phthalates on sexual behavior refers to the phenomenon where the effect of these compounds on sexual development and/or behavior does not show a linear relationship. Phthalates in specific concentrations can modulate hormonal balance and affect the reproductive system. However, their effects show a non-linear pattern at different doses, with lower and/or higher concentrations producing different results. In addition, since humans and other animals are exposed to various types of phthalates and other endocrine disruptors at the same time, and their effects are often contradictory, it is a challenge to investigate exact mechanisms of action.

The study’s sample size of 68 female university students and non-validated questionnaire may limit the generalizability of findings. Also, a validated psychometric tool for measuring the intensity of female pleasure, the Orgasmometer, was not utilized in this study. While demographic factors are considered, other potential confounders, such as lifestyle factors and psychological variables, are not adequately addressed, which are crucial for assessing subjective sexual function. The cross-sectional design of the study limits the ability to establish causal relationships between phthalate exposure and sexual function. Conversely, the strengths of our study lie in the convergence of information related to perinatal androgenization, cumulative phthalate exposure, and subjective sexual function obtained from the same cohort of reproductive-age females. In the context of sexuality and toxicology, the “butterfly effect” observed in our study means that even minor exposures to certain chemicals or toxins can potentially lead to substantial and sometimes unpredictable effects on biological systems. A seemingly insignificant exposure to a toxic substance, even at low concentrations, could initiate a cascade of biological responses within an organism. These responses may include biochemical reactions, cellular damage, alterations in gene expression, and ultimately, adverse health effects. Moreover, the effects of toxic exposures can propagate through complex biological networks, influencing various organs, tissues, and physiological processes. Over time, these initial exposures may result in long-term health consequences, including diminished sexual functions. Therefore, understanding the butterfly effect in toxicology underscores the importance of considering not only the direct effects of toxic substances but also potential indirect and cumulative impacts of potential confounding factors (such as prenatal androgenization) on biological systems. This perspective highlights the need for comprehensive risk assessments and precautionary measures to mitigate the potential harm associated with exposure to toxic agents, even at seemingly insignificant levels.

Future studies of phthalate exposure on human sexual function should include specific validated measures of sexual desire in both females and males. Studies should also examine objective measures of genital and cognitive sexual arousal to visual sexual stimuli using psychophysiological techniques (photoplethysmography, galvanic skin response, EEG) and eye-tracking, in addition to subjective measures of sexual arousal, desire, and satisfaction, along with sexual excitation and inhibition [40–42]. Future studies should also monitor plasma levels of T, E2, P4, cortisol, and steroid hormone binding globulins, along with gonadotropins and corticotropins, when behavioral assessments of sexual function are made. It would also be useful to contrast different EDC effects on sexual function and to determine the relative contribution of estrogenic or anti-androgenic actions.

## Supplementary information

Supplementary material

## Data Availability

All data are available on request from the corresponding author. Data cannot be shared publicly due to the risk of violating privacy.
