# Supplementary material for: Endocrine effect of phthalate metabolites and a butterfly effect of prenatal exposure to androgens on qualitative aspects of female sexual response- an initial survey
Source: Int J Impot Res. 2024 May 28;37(4):329–37. doi: 10.1038/s41443-024-00919-1 (PMC12043500; doi:10.1038/s41443-024-00919-1)
Supplement: Supplementary file 1 — Supplementary material [file 41443_2024_919_MOESM1_ESM.pdf]

Supplement 1

Endocrine effect of phthalate metabolites and a butterfly effect of prenatalexposure to androgens on qualitative aspects of female sexual response- an initial survey

GENERAL QUESTIONNAIRE OF SUBJECTIVE SEXUAL FUNCTION

A. Please indicate the frequency of your sexual intercourse in the last month.

The answer	Scale	Value
Not even once	*0*	*0*
Once a month	*1*	*0.143*
Once every 2 weeks	*2*	*0.286*
Once a week	*3*	*0.429*
Twice a week	*4*	*0.572*
3-4 times a week	*5*	*0.715*
Once a day	*6*	*0.858*
More often than once a day	*7*	*1.001*

B. How often have you masturbated in the last month?

The answer	Scale	Value
Not even once	*0*	*0*
Once a month	*1*	*0.143*
Once every 2 weeks	*2*	*0.286*
Once a week	*3*	*0.429*
Twice a week	*4*	*0.572*
3-4 times a week	*5*	*0.715*
Once a day	*6*	*0.858*
More often than once a day	*7*	*1.001*

C. "My current sexual desire (excitement) is strong."

The answer	Scale	Value
I completely disagree	*0*	*0*
I rather disagree	*1*	*0.25*
Is adequate (normal) as usual	*2*	*0.5*
I rather agree	*3*	*0.75*
I totally agree	*4*	*1*

D. How often do you have sexual fantasies?

The answer	Scale	Value
Not even once	*0*	*0*
Less than once a month	*1*	*0.25*
Once a month to 2 times a week	*2*	*0.5*
More than 2 times a week	*3*	*0.75*
Daily	*4*	*1*

E. Please express your satisfaction/dissatisfaction with your sexual arousal.

The answer	Scale	Value
I don't feel any pleasure or excitement	*0*	*0*
I experience a faint excitement	*1*	*0.25*
I feel a slight excitement	*2*	*0.5*

I feel a strong excitement	*3*	*0.75*
I feel a very strong excitement	*4*	*1*

F. How often do you have a desire for sexual satisfaction?

**The answer**	**Scale**	**Value**
Not even once	*0*	*0*
Less than once a month	*1*	*0.25*
Once a month to 2 times a week	*2*	*0.5*
More than 2 times a week	*3*	*0.75*
Daily	*4*	*1*

G. Are you interested in books, movies, websites, or drawings with a sexual theme?

**The answer**	**Scale**	**Value**
Not even once	*0*	*0*
Less than once a month	*1*	*0.25*
Once a month to 2 times a week	*2*	*0.5*
More than 2 times a week	*3*	*0.75*
Daily	*4*	*1*

H. If you were to compare yourself to your peers, how intense is your sexual desire?

**The answer**	**Scale**	**Value**
Definitely weaker	*0*	*0*
Weaker	*1*	*0.25*
Approximately the same	*2*	*0.5*
Stronger	*3*	*0.75*
Definitely stronger	*4*	*1*

Supplement 2

Supplementary table 1: Results of hierarchical linear regression of the association between score of sexuality and phthalate metabolites adjusted for digit ratio (2D:4D)

**Model: Score of sexuality**		***ß***	**95% CI**		***p* (coefficient)**	**r^2 (model)**	***p* (model)**
1	MMP	-0.056	-0.292	0.199	0.656	0.003	0.656
2	MMP	-0.051	-0.299	0.196	0.680	0.019	0.539
	Digit ratio (2D:4D)	-0.127	-0.372	0.123	0.310		
1	MEP	0.069	-0.180	0.310	0.577	0.005	0.577
2	MEP	0.067	-0.181	0.314	0.590	0.021	0.507
	Digit ratio (2D:4D)	-0.127	-0.372	0.123	0.308		
1	MiBP	-0.298	-0.535	-0.066	0.014	0.089	0.014
2	MiBP	-0.299	-0.536	-0.063	0.014	0.106	0.028
	Digit ratio (2D:4D)	-0.131	-0.365	0.107	0.272		
1	OH-MiBP	-0.070	-0.321	0.169	0.575	0.005	0.575
2	OH-MiBP	-0.072	-0.320	0.174	0.560	0.022	0.495
	Digit ratio (2D:4D)	-0.130	-0.375	0.119	0.297		
1	MnBP	-0.063	-0.329	0.161	0.613	0.004	0.613
2	MnBP	-0.066	-0.314	0.181	0.595	0.021	0.509
	Digit ratio (2D:4D)	-0.130	-0.375	0.119	0.297		
1	OH-MnBP	-0.301	-0.542	-0.075	0.013	0.091	0.013
2	OH-MnBP	-0.299	-0.536	-0.063	0.014	0.106	0.028
	Digit ratio (2D:4D)	-0.123	-0.358	0.115	0.304		
1	MBzP	-0.209	-0.463	0.016	0.090	0.044	0.09
2	MBzP	-0.203	-0.446	0.040	0.100	0.057	0.15
	Digit ratio (2D:4D)	-0.118	-0.358	0.127	0.336		
1	MCHP	-0.209	-0.445	0.036	0.090	0.044	0.09
2	MCHP	-0.205	-0.448	0.037	0.096	0.058	0.146
	Digit ratio (2D:4D)	-0.122	-0.363	0.122	0.320		
1	MEHP	-0.181	-0.434	0.048	0.142	0.033	0.142
2	MEHP	-0.167	-0.414	0.080	0.181	0.044	0.238
	Digit ratio (2D:4D)	-0.106	-0.349	0.144	0.395		
1	OH-MEHP	-0.181	-0.455	0.025	0.144	0.033	0.144
2	OH-MEHP	-0.196	-0.439	0.049	0.113	0.055	0.166
	Digit ratio (2D:4D)	-0.149	-0.389	0.099	0.227		
1	oxo-MEHP	-0.236	-0.502	-0.028	0.055	0.056	0.055
2	oxo-MEHP	-0.243	-0.483	-0.002	0.048	0.075	0.082
	Digit ratio (2D:4D)	-0.140	-0.378	0.103	0.249		
1	cx-MEPP	-0.257	-0.523	-0.052	0.036	0.066	0.036
2	cx-MEPP	-0.261	-0.500	-0.022	0.033	0.085	0.059
	Digit ratio (2D:4D)	-0.138	-0.374	0.104	0.254		

1	McMHP	-0.362	-0.618	-0.166	0.003	0.131	0.003
2	McMHP	-0.359	-0.590	-0.128	0.003	0.145	0.007
	Digit ratio (2D:4D)	-0.117	-0.346	0.116	0.316		
1	OH-MiNP	-0.007	-0.266	0.226	0.952	0	0.952
2	OH-MiNP	-0.014	-0.262	0.234	0.911	0.017	0.584
	Digit ratio (2D:4D)	-0.129	-0.375	0.121	0.302		
1	oxo-MiNP	-0.006	-0.278	0.213	0.960	0	0.96
2	oxo-MiNP	-0.010	-0.258	0.238	0.936	0.017	0.585
	Digit ratio (2D:4D)	-0.129	-0.374	0.122	0.303		
1	cx-MiNP	0.052	-0.222	0.270	0.677	0.003	0.677
2	cx-MiNP	0.058	-0.190	0.306	0.642	0.02	0.527
	Digit ratio (2D:4D)	-0.131	-0.376	0.119	0.294		

Notes: 2D-length of the right index finger, 4D-length of the right ring finger, β-standardized coefficient beta, CI-confidence interval, cx-MEPP—mono(2-ethyl-5-carboxypentyl) phthalate, cx-MiNP— mono-carboxy-isononyl phthalate, MBzP—monobenzyl phthalate, MCHP—monocyclohexyl phthalate, MEP—monoethyl phthalate, MiBP—mono-iso-butyl phthalate, MMP—monomethyl phthalate, MnBP—mono-n-butyl phthalate, OH-MEHP—mono(2-ethyl-5-hydroxyhexyl) phthalate, OH-MiBP—mono(hydroxy-iso- butyl) phthalate, OH-MiNP— mono-hydroxy-isononyl phthalate, OH-MnBP—mono(hydroxy-n-butyl) phthalate, oxo-MEHP—mono(2-ethyl-5-oxohexyl) phthalate, oxo-MiNP— mono-oxo-isononyl phthalate, p-value of statistical significance, r-correlation coefficient; Hierarchical multiple regression analysis was used

Supplementary table 2: Results of hierarchical linear regression of the association between score of sexuality and phthalate metabolites adjusted for length of right index finger (2D)

**Model: Score of sexuality**		***β***	**95% CI**		***p* (coefficient)**	**r^2 (model)**	***p* (model)**
1	MMP	-0.056	-0.292	0.199	0.656	0.003	0.656
2	MMP	-0.062	-0.313	0.188	0.622	0.007	0.807
	2D	-0.060	-0.311	0.190	0.632		
1	MEP	0.069	-0.180	0.310	0.577	0.005	0.577
2	MEP	0.073	-0.176	0.322	0.560	0.008	0.769
	2D	-0.058	-0.307	0.191	0.643		
1	MiBP	-0.298	-0.535	-0.066	0.014	0.089	0.014
2	MiBP	-0.296	-0.535	-0.056	0.016	,089	0.05
	2D	-0.022	-0.261	0.218	0.857		
1	OH-MiBP	-0.070	-0.321	0.169	0.575	0.005	0.575
2	OH-MiBP	-0.072	-0.321	0.177	0.565	0.008	0.773
	2D	-0.056	-0.305	0.193	0.653		
1	MnBP	-0.063	-0.329	0.161	0.613	0.004	0.613
2	MnBP	-0.059	-0.309	0.191	0.640	0.006	0.817
	2D	-0.048	-0.298	0.201	0.700		
1	OH-MnBP	-0.301	-0.542	-0.075	0.013	0.091	0.013
2	OH-MnBP	-0.299	-0.538	-0.061	0.015	0.092	0.045
	2D	-0.037	-0.275	0.201	0.756		

1	MBzP	-0.209	-0.463	0.016	0.090	0.044	0.09
2	MBzP	-0.217	-0.462	0.028	0.082	0.049	0.198
	2D	-0.076	-0.321	0.169	0.538		
1	MCHP	-0.209	-0.445	0.036	0.090	0.044	0.09
2	MCHP	-0.205	-0.451	0.040	0.100	0.044	0.233
	2D	-0.029	-0.275	0.217	0.813		
1	MEHP	-0.181	-0.434	0.048	0.142	0.033	0.142
2	MEHP	-0.181	-0.426	0.065	0.146	0.036	0.314
	2D	-0.052	-0.297	0.194	0.676		
1	OH-MEHP	-0.181	-0.455	0.025	0.144	0.033	0.144
2	OH-MEHP	-0.177	-0.425	0.072	0.161	0.033	0.34
	2D	-0.025	-0.273	0.224	0.845		
1	oxo-MEHP	-0.236	-0.502	-0.028	0.055	0.056	0.055
2	oxo-MEHP	-0.234	-0.477	0.009	0.059	0.057	0.151
	2D	-0.042	-0.285	0.201	0.732		
1	cx-MEPP	-0.257	-0.523	-0.052	0.036	0.066	0.036
2	cx-MEPP	-0.255	-0.496	-0.013	0.039	0.068	0.106
	2D	-0.043	-0.284	0.198	0.724		
1	McMHP	-0.362	-0.618	-0.166	0.003	0.131	0.003
2	McMHP	-0.361	-0.594	-0.127	0.003	0.132	0.011
	2D	-0.029	-0.262	0.204	0.803		
1	OH-MiNP	-0.007	-0.266	0.226	0.952	0	0.952
2	OH-MiNP	-0.004	-0.262	0.234	0.974	0.003	0.912
	2D	-0.053	-0.375	0.121	0.672		
1	oxo-MiNP	-0.006	-0.278	0.213	0.960	0	0.96
2	oxo-MiNP	-0.006	-0.255	0.243	0.963	0.003	0.912
	2D	-0.053	-0.303	0.196	0.670		
1	cx-MiNP	0.052	-0.222	0.270	0.677	0.003	0.677
2	cx-MiNP	0.056	-0.193	0.306	0.655	0.006	0.825
	2D	-0.058	-0.307	0.192	0.646		

Notes: 2D-length of the right index finger, 4D-length of the right ring finger, β- standardized coefficient beta, CI-confidence interval, cx-MEPP—mono(2-ethyl-5-carboxypentyl) phthalate, cx-MiNP— mono-carboxy-isononyl phthalate, MBzP—monobenzyl phthalate, MCHP—monocyclohexyl phthalate, MEP—monoethyl phthalate, MiBP—mono-iso-butyl phthalate, MMP—monomethyl phthalate, MnBP—mono-n-butyl phthalate, OH-MEHP—mono(2-ethyl-5-hydroxyhexyl) phthalate, OH-MiBP—mono(hydroxy-iso- butyl) phthalate, OH-MiNP— mono-hydroxy-isononyl phthalate, OH-MnBP—mono(hydroxy-n-butyl) phthalate, oxo-MEHP—mono(2-ethyl-5-oxohexyl) phthalate, oxo-MiNP— mono-oxo-isononyl phthalate, p-value of statistical significance, r-correlation coefficient; Hierarchical multiple regression analysis was used

Supplementary table 3: Results of hierarchical linear regression of the association between score of sexuality and phthalate metabolites adjusted for length of right ring finger (4D)

**Model: Score of sexuality**		***β***	**95% CI**		***p* (coefficient)**	**r^2 (model)**	***p* (model)**
1	MMP	-0.056	-0.292	0.199	0.656	0.003	0.656

2	MMP	-0.052	-0.304	0.200	0.682	0.004	0.886
	4D	0.026	-0.225	0.278	0.835		
1	MEP	0.069	-0.180	0.310	0.577	0.005	0.577
2	MEP	0.068	-0.182	0.317	0.590	0.006	0.833
	4D	0.030	-0.220	0.279	0.814		
1	MiBP	-0.298	-0.535	-0.066	0.014	0.089	0.014
2	MiBP	-0.302	-0.541	-0.064	0.014	0.092	0.045
	4D	0.057	-0.182	0.296	0.636		
1	OH-MiBP	-0.070	-0.321	0.169	0.575	0.005	0.575
2	OH-MiBP	-0.069	-0.318	0.181	0.584	0.006	0.829
	4D	0.031	-0.218	0.280	0.803		
1	MnBP	-0.063	-0.329	0.161	0.613	0.004	0.613
2	MnBP	-0.066	-0.316	0.184	0.599	0.005	0.839
	4D	0.039	-0.211	0.289	0.756		
1	OH-MnBP	-0.301	-0.542	-0.075	0.013	0.091	0.013
2	OH-MnBP	-0.302	-0.540	-0.065	0.014	0.093	0.045
	4D	0.040	-0.197	0.278	0.735		
1	MBzP	-0.209	-0.463	0.016	0.090	0.044	0.09
2	MBzP	-0.209	-0.456	0.039	0.097	0.044	0.24
	4D	0.002	-0.245	0.249	0.987		
1	MCHP	-0.209	-0.445	0.036	0.090	0.044	0.09
2	MCHP	-0.212	-0.457	0.032	0.088	0.046	0.222
	4D	0.048	-0.196	0.293	0.694		
1	MEHP	-0.181	-0.434	0.048	0.142	0.033	0.142
2	MEHP	-0.180	-0.427	0.067	0.151	0.033	0.341
	4D	0.014	-0.233	0.261	0.911		
1	OH-MEHP	-0.181	-0.455	0.025	0.144	0.033	0.144
2	OH-MEHP	-0.196	-0.477	0.054	0.122	0.038	0.29
	4D	0.075	-0.176	0.325	0.553		
1	oxo-MEHP	-0.236	-0.502	-0.028	0.055	0.056	0.055
2	oxo-MEHP	-0.238	-0.481	0.005	0.054	0.058	0.149
	4D	0.045	-0.197	0.288	0.711		
1	cx-MEPP	-0.257	-0.523	-0.052	0.036	0.066	0.036
2	cx-MEPP	-0.258	-0.499	-0.017	0.037	0.068	0.107
	4D	0.042	-0.199	0.284	0.727		
1	McMHP	-0.362	-0.618	-0.166	0.003	0.131	0.003
2	McMHP	-0.363	-0.596	-0.131	0.003	0.133	0.01
	4D	0.039	-0.193	0.272	0.736		
1	OH-MiNP	-0.007	-0.266	0.226	0.952	0	0.952
2	OH-MiNP	-0.010	-0.260	0.240	0.936	0.001	0.961
	4D	0.034	-0.216	0.285	0.784		

1	oxo-MiNP	-0.006	-0.278	0.213	0.960	0	0.96
2	oxo-MiNP	-0.006	-0.256	0.243	0.961	0.001	0.963
	4D	0.034	-0.216	0.283	0.788		
1	cx-MiNP	0.052	-0.222	0.270	0.677	0.003	0.677
2	cx-MiNP	0.051	-0.198	0.301	0.682	0.004	0.886
	4D	0.033	-0.216	0.282	0.793		

Notes: 2D-length of the right index finger, 4D-length of the right ring finger, β- standardized coefficient beta, CI-confidence interval, cx-MEPP—mono(2-ethyl-5-carboxypentyl) phthalate, cx-MiNP— mono-carboxy-isononyl phthalate, MBzP—monobenzyl phthalate, MCHP—monocyclohexyl phthalate, MEP—monoethyl phthalate, MiBP—mono-iso-butyl phthalate, MMP—monomethyl phthalate, MnBP—mono-n-butyl phthalate, OH-MEHP—mono(2-ethyl-5-hydroxyhexyl) phthalate, OH-MiBP—mono(hydroxy-iso- butyl) phthalate, OH-MiNP— mono-hydroxy-isononyl phthalate, OH-MnBP—mono(hydroxy-n-butyl) phthalate, oxo-MEHP—mono(2-ethyl-5-oxohexyl) phthalate, oxo-MiNP— mono-oxo-isononyl phthalate, p-value of statistical significance, r-correlation coefficient; Hierarchical multiple regression analysis was used
